# Roundtable discussion: how lessons learned from HIV can inform the global response to viral hepatitis

**DOI:** 10.1186/1471-2334-14-S6-S18

**Published:** 2014-09-19

**Authors:** Jeffrey V Lazarus, Jens Lundgren, Jordi Casabona, Lucas Wiessing, Catharina Matheï, Peter Vickerman, Maria Prins, Mirjam Kretzschmar, Maria Kantzanou, Isabelle Giraudon, Marica Ferri, Paul Griffiths, Magdalena Harris, Margaret Walker, Lilyana Chavdarova, Eberhard Schatz, Katrin Schiffer, John Peter Kools, Jason Farell, Luís Mendão

**Affiliations:** 1 CHIP, Centre for Health and Infectious Disease Research and WHO Collaborating Centre on HIV and Viral Hepatitis, Rigshospitalet, University of Copenhagen, Copenhagen, Denmark; 2 Epidemiological Center for HIV/AIDS/STI of Catalonia (CEEISCAT), Barcelona, Spain; 3 European Monitoring Centre for Drugs and Drug Addiction (EMCDDA), Lisbon, Portugal; 4 Department of Public Health and Primary Care, KU Leuven, Leuven, Belgium; 5 London School of Hygiene and Tropical Medicine (LSHTM), London, UK; 6 School of Social and Community Medicine, University of Bristol, Bristol, UK; 7 Public Health Service, Cluster Infectious Diseases, Department of Research, Amsterdam, the Netherlands; 8 Center for Infection and Immunity (CINIMA), Academic Medical Center, Amsterdam, the Netherlands; 9 Julius Centre for Health Sciences and Primary Care, University Medical Centre Utrecht, Utrecht, the Netherlands; 10 Centre for Infectious Disease Control, National Institute for Public Health and the Environment, Bilthoven, the Netherlands; 11 National Reference Centre for Retroviruses, Laboratory of Hygiene, Epidemiology and Medical Statistics, University of Athens Medical School, Athens, Greece; 12 London School of Hygiene and Tropical Medicine (LSHTM), London, UK; 13 European Liver Patients’ Associaton, B-3800 Sint-Truiden, Belgium; 14 Correlation – European Network Social Inclusion and Health, Amsterdam, the Netherlands; 15 G.A.T. – Grupo Português de Activistas sobre Tratamentos de VIH/SIDA – Pedro Santos, Portugal; 16 European AIDS Treatment Group, B-1050 Brussels, Belgium

## Introduction

*By **Jeffrey V Lazarus***.

In the last few years, as viral hepatitis has become a more prominent issue on national and international health agendas, talk of "learning from HIV" has become quite common. It is understandable for researchers, policy-makers and activists to make this link, given the notable commonalities between HIV, hepatitis B and hepatitis C. All are life-threatening blood-borne viruses that affect hundreds of millions of people worldwide. All can remain asymptomatic for many years, making it difficult to promote awareness of their danger. Ignorance about all of the major forms of viral hepatitis, particularly B and C, understandably reminds many people who are working to confront this issue of the early years of the AIDS epidemic, when a combination of misinformation and unwillingness to candidly address stigmatised behaviours and social inequalities led to a widespread failure to formulate a cohesive public health response.

Other parallels might be elucidated, and there is certainly good reason to think of the global HIV, hepatitis B and hepatitis C epidemics as having similar trajectories in some regards. Nonetheless, the differences between HIV and viral hepatitis – in terms of how these varied diseases manifest both biologically and socially – should make us wary of oversimplifying the connection.

As discussions about hepatitis policies and strategies gain momentum at the national and global level, it is becoming increasingly important to articulate the "lessons learned" from the HIV field. Pertinent issues include disease prevention measures, testing and early diagnosis, the scale-up of treatment, and barriers to service uptake and retention in care. Key social and political factors associated with viral hepatitis call to mind other possible lessons relating to the value of civil society engagement, leadership and governance issues, resource mobilization including innovative financing, the causes and consequences of stigma, and the role of social science in addressing health threats.

This is clearly a rich and important realm of inquiry – and it is time to move beyond the general calls to learn from HIV and begin to systematically formulate specific lessons. As a preliminary step, the following roundtable presents the insights of a range of experts who were invited to reflect on how experiences from the global response to HIV might inform the global response to viral hepatitis.

Kicking off the discussion is Jens Lundgren, a renowned HIV researcher, head of the new WHO Collaborating Centre on HIV and Viral Hepatitis and co-founder of the HIV in Europe Initiative. His perspective is much broader than that of a medical doctor gazing at his own disease. In the next two pieces, Jordi Casabona of the Epidemiological Center for HIV/AIDS/STI of Catalonia and Lucas Wiessing of the European Monitoring Centre for Drugs and Drug Addiction (and colleagues) consider surveillance and monitoring issues. Sounding a cautionary note about the “discourse of hope and expectation” associated with hepatitis C treatment, Magdalena Harris, a qualitative researcher at the London School of Hygiene and Tropical Medicine, critically reflects on treatment as prevention.

The European Liver Patients Association raises a number of key issues including how price reductions for HIV treatment should inspire stakeholders to find ways of making the newest hepatitis C medications more affordable. Leaders of the Correlation Network’s Hepatitis C Initiative, which commissioned this supplement and which is holding the first European Conference on Hepatitis and Injecting Drug Use (October 2104), identify key actions to address hepatitis based on lessons learned from HIV, with a focus on people who inject drugs. Finally, in keeping with the growing global emphasis on people-centred health systems, Luís Mendão of Portugal, a leader of the European AIDS Treatment Group, recounts what it is like to live with both HIV and hepatitis.

## Much to learn and adapt from over three decades of HIV

*By **Jens Lundgren**, Director of CHIP, Centre for Health and Infectious Disease Research and WHO Collaborating Centre on HIV and Viral Hepatitis*.

HIV, hepatitis B and hepatitis C share many features including common modes of transmission and the availability of effective treatment, albeit only curative for hepatitis C. As effective treatment was developed earlier for HIV (1996) than for hepatitis B and hepatitis C (2004 and 2014, respectively), some important lessons on the public health aspects of providing HIV therapy to those in need may be informative to guide the public health response to the two types of viral hepatitis.

A major dilemma in the HIV public health response has been, and to some extent still is, the fact that no ideal strategy has been developed for testing the population to identify those infected. As a consequence, a large number of HIV-positive people remain undiagnosed. As the infection remains asymptomatic early on, it is no surprise that most undiagnosed people are in the early stages of infection, and only seek and receive a diagnosis when the infection has progressed and the patient develops AIDS or other severe manifestations of the immunodeficiency caused by the virus. These late presenters still constitute some 50% of newly diagnosed HIV patients across Europe [1]. Agreement on a definition of late presenters has been an important tool incorporated into surveillance structures to understand to what extent a large undiagnosed population remains a problem. A definition of chronic viral hepatitis-infected persons who present late for care remains to be established.

Along these lines, the estimation of the number of undiagnosed people constitutes core knowledge that should be helping to guide public health responses. Given that those undiagnosed are not picked up by routine surveillance, the critical missing link in the HIV response was to use surveillance information as the basis for mathematical models that can provide a reasonable estimate of the number of infected and undiagnosed people living with HIV in a given population. Multiple approaches on how to resolve this are underway [2]. There is an urgent need to initiate similar activities so that the number of undiagnosed individuals with hepatitis B and hepatitis C can be more reliably estimated.

Testing for HIV is a diagnostic technique similar to other diagnostic techniques performed in people who seek contact with the health system. The fact of the matter is, however, that most HIV tests are done in specialised units, not by general practitioners. Research in more recent years has demonstrated that many of the undiagnosed people living with HIV come into contact with the health system several times before they are diagnosed with HIV. Each of these visits obviously constitutes a missed opportunity to catch the person earlier in the course of the infection. Mapping efforts are underway to determine the conditions these people had when approaching the health system [3]. The first set of guidelines regarding which conditions should prompt any health professional to offer an HIV test was released in 2012 (http://www.hpa.org.uk/webc/HPAwebFile/HPAweb_C/1317133743551), and the number of conditions continues to expand (http://hiveurope.eu/Ongoing-Projects/HIDES/HIDES-2). This author’s personal view is that the situation for undiagnosed hepatitis B and hepatitis C resembles that for HIV; future research should document whether this view is correct. Additionally, more proactive community-based testing is being implemented for HIV, and the challenges associated with linking those diagnosed with HIV to the health system are being explored. The realisation that a comprehensive testing approach utilising multiple and diverse approaches tailored to the local situation should be the standard for all three viral infections.

Health systems have adapted their approach to offering HIV care. The patient community has been an active partner in this adaptation process. Community clinics and shared care models are being implemented. Linkage to and retention in care are both critical indicators of how effectively the health system is providing the care required. The concept of understanding the “treatment cascade” in the setting you are responsible for is becoming a best-practice standard, with recognition that concerted actions should be implemented to understand and resolve barriers to suboptimal performance. Mapping exercises of the treatment cascade for hepatitis B and hepatitis C will be critical to guide the public health response and to ensure care for those in need of effective therapy.

Much of the progress in refining the public health response to HIV has been seen in the last decade. An important lesson from the process is that none of the key stakeholders can do this by themselves. As such, civil society, health professionals and policy-makers need to collaborate and create a collegial atmosphere for the collaboration to be fruitful. Each of these constituencies brings important knowledge and abilities to the process. This is also true when considering the challenges associated with hepatitis B and hepatitis C.

Above are some examples of progress made within the HIV field that can be considered for modification and adaption to guide a comprehensive and rational public health response to viral hepatitis. That said, it is important to also stress that whereas much progress in the public health response to HIV has been achieved in the last decade, we have by no means a perfect system. Further refining the public health response to HIV will require overcoming considerable challenges. But at least we are now able to formulate a series of best-practice examples, and in doing so, identify those that hold up well and those where there is room for improvement.

## Surveilling infectious diseases

*By **Jordi Casabona**, Director of the Epidemiological Center for HIV/AIDS/STI of Catalonia (CEEISCAT)*.

The aim of surveillance of infectious diseases is to improve the prevention and control of these conditions by public health authorities as well as to inform clinical services to better deliver both prevention and treatment programmes. Therefore, surveillance approaches and strategies evolve in accordance with the knowledge we have on the particular agent and the natural history of the disease. They also evolve in response to the diagnostic and therapeutic tools available. For instance, the identification of HIV in 1983 and the development of the first serological test in 1985 allowed for the design and implementation of bio-behavioural studies among specific sentinel populations. When in 1996 combination antiretroviral therapy proved to be highly effective, intense screening programmes to detect people living with HIV were put in place; when later on it was demonstrated that initiating treatment earlier could improve clinical outcomes, early diagnosis strategies were developed to decrease the number of HIV-infected people who do not know they are infected.

All of this required surveillance strategies to evolve over time to incorporate new methods and variables. While HIV knowledge has been steadily accumulating for the last 20 years, information about hepatitis C treatment has increased significantly only during the last few years. Information systems on HIV have been expanding in scope and complexity over a long period of time, but hepatitis C-related information systems are scarce. The recent identification of new hepatitis C treatments than can in most cases cure the patient forces public health administrators to draw on the experience of monitoring HIV and its diagnosis and treatment in order to rapidly improve the coverage and quality of hepatitis-related epidemiological, diagnostic and treatment efforts, for instance incorporating hepatitis C data-gathering into all formal HIV surveillance and observational studies.

## Tackling hepatitis C among PWID in Europe: what can we learn from HIV?

*By **Lucas Wiessing**, **Catharina Matheï**, **Peter Vickerman**, **Maria Prins**, **Mirjam Kretzschmar**, **Maria Kantzanou**, **Isabelle Giraudon**, **Marica Ferri** and **Paul Griffiths***.

Chronic infection with the hepatitis C virus (HCV) affects about 160 million people worldwide causing over 350,000 deaths per year [4-6]. In Europe, it is highly concentrated in people who inject drugs (PWID) due to transmission related to the sharing of injecting equipment, and this is now a key at-risk group [7-9].

Recent new treatments potentially could have a large impact on HCV in PWID both in preventing liver disease and death, as well as further transmission, but their high cost may hamper full implementation. Also, PWID often have less access to healthcare due to stigma, discrimination and repressive drug use policies [10]. While coverage of traditional HCV treatments remains low in PWID [11], despite recent reviews suggesting they achieve similar cure rates, it remains to be seen if a large scale-up with the new HCV treatments in PWID is possible, similar to the universal HIV treatment access policies adopted in the 1990s.

The EMCDDA monitors prevalence data of HIV, HBV and HCV among PWID across 30 European countries, enabling the tracking of trends in infection levels and the identification of countries and regions at risk [7,12,13]. It also monitors levels and trends in intervention coverage (opioid substitution treatment and needle and syringe programmes) [14] and population sizes of PWID. Additionally, behavioural indicators (e.g. needle sharing, HIV/HCV testing) have recently been developed following a second-generation HIV surveillance framework.

Modelling work initiated by and undertaken in collaboration with the EMCDDA has suggested that HCV prevalence may provide an important indicator of injecting risks among PWID populations and that it may predict the risk of HIV outbreaks in those populations [15,16]. This appears to have been confirmed in recent HIV outbreaks in Europe, with increases in HCV prevalence preceding the increases in HIV transmission [12].

Based on methodology developed by the EMCDDA, HCV prevalence data have thus been used to assess the risks of HIV outbreaks across Europe, in combination with other risk indicators and indicators of intervention coverage [17,18]. Several countries show increasing HCV prevalence among PWID, suggesting an increased risk of HIV transmission and potentially new HIV epidemics in this population [12,13]. In addition, it has shown the potential impact of antiretroviral therapy as prevention on HIV incidence in populations of PWID.

Recently, the EMCDDA carried out a systematic review of the availability of key data to guide the scaling up of HCV treatment in PWID across Europe, as a complement to the indicators routinely monitored (e.g. HIV and HCV prevalence, PWID population size, intervention coverage) [11]. The study indicated that data availability in several areas of importance for HCV in PWID (e.g. incidence, chronicity, diagnosis, treatment access and burden of disease) is limited, whereas the data that are available suggest low rates of HCV diagnosis and HCV treatment entry among PWID (higher data availability was found for genotypes and HIV co-infection).

Based on the experience so far, three points can be mentioned as conditions for effective policies regarding HCV in PWID in Europe:

1. Given high HCV transmission coupled with low levels of diagnosis and treatment among PWID, as well as rapid developments in treatment effectiveness, the case for full access to antiviral treatment for PWID is becoming as pertinent as it has been for HIV since the mid-1990s. This includes a need for renegotiation of treatment prices at the EU level as current prices (up to 63 000 euros for one treatment) are prohibitive, especially where HIV is already incurring a large cost burden. EMCDDA, in collaboration with key national, European and international partners, is routinely reporting on trends in HCV and HIV epidemiology and prevention coverage among PWID. However, HCV treatment coverage monitoring is not in place and needs to be implemented, while existing monitoring systems, often still based on partial data, need to be strengthened and consolidated.

2. The combination of observational (cohort and bio-behavioural) and modelling studies has proven essential for our understanding of epidemiology, intervention best practice and policy options with regard to both HCV and HIV in PWID [19, 20]. A stronger investment in multidisciplinary studies using country-specific data with the aim to support national policies is greatly needed.

3. PWID have many specific needs that extend beyond HCV, including for example shared risk factors for HIV and other infectious diseases, a high risk of death and often serious social, somatic and psychiatric co-morbidity and legal problems. These call for multifaceted and integrated interventions, one of which is the treatment of HCV infection. Sound public health policies need to make full use of the existing expertise and specialised data systems with regard to PWID in Europe in close collaboration and collegial exchange with the generalised public health expertise and infrastructures at the national and international levels.

## A discourse of hope? Avoiding pitfalls with ‘treatment as prevention’ for hepatitis C

*By **Magdalena Harris** Lecturer, London School of Hygiene and Tropical Medicine*.

Current innovations in hepatitis C drug development are reflected in a discourse of hope and expectation, with references to “viral elimination” and “treatment as prevention” increasingly noted in policy and academic literature [21]. The concept of treatment as prevention (TasP), while familiar to the HIV sector, is nascent in relation to hepatitis C. While this discourse reflects an increased sense of treatment possibility, critical reflection on its implications is warranted.

The linkage of hepatitis C treatment with prevention has been occasioned by the advent of efficacious direct-acting antivirals coupled with modelling work that illustrates the impact of treating hepatitis C-infected people who inject drugs (PWID) on the prevalent pool of the virus. This population-based approach has implications for clinical treatment decision-making, which is generally based on an individualised assessment of potential treatment responsiveness and stage of disease progression. Treatment as a prevention strategy requires the scale-up of treatment for people who are currently injecting – an especially important point in the context of the routine denial of treatment for this priority population.

What, however, are the potential limitations of a TasP framework? What are the conditions under which it may be workable? Looking to the HIV field provides some insights:

1. *TasP must not undermine prevention as prevention.* Scholars such as Nguyen et al [22] have lamented the impact of TasP in re-medicalising the HIV epidemic and subordinating primary prevention funding to a treatment agenda. Harm reduction initiatives for PWID such as needle and syringe programmes and opioid substitution therapy are already fragile: politically unpopular (or prohibited) and under-resourced. TasP is unsustainable without a concomitant commitment to the up-scaling and resourcing of harm reduction or “prevention as prevention” initiatives.

2. *Prevention initiatives must be community-owned and community-led.* In order for prevention initiatives – including those involving hepatitis C treatment – to be successful, they must be supported and endorsed by the affected community. TasP is premised on a population level of analysis – the theoretical modelling work on which it is based is divorced from the grounded experience of people who navigate hepatitis C and HIV risk in their daily lives. The acceptability and uptake of interventions depend on engagement with this experience and the meaningful involvement of affected communities in their implementation. This is particularly crucial in relation to PWID, for whom criminalisation and systemic discrimination place fundamental barriers to service access.

3. *TasP must not undermine social structural interventions.* For many PWID, hepatitis C is not a priority – and may still not be even with the advent of more efficacious treatments. Social structural barriers to treatment access are reflected in peoples’ lives more broadly – for many PWID, issues such as homelessness, poverty and the threat of incarceration take precedence over more long-term concerns such as hepatitis C. As Nguyen et al comment, “treatment is not a substitute to the removal of the vulnerabilities that place people at risk of infection in the first place” [22]. In the absence of resources for community empowerment and interventions tackling stigma and inequality, TasP is unsustainable and potentially unethical.

4. *Treatment is a human rights issue.* A population-based impetus to increase treatment access and uptake among PWID can place an unwelcome onus on already-marginalised individuals to undertake treatment for which they may not be ready or willing. In the absence of needed resources, TasP threatens to locate responsibility for low treatment uptake with the affected community rather than with the social institutions and conditions generative of treatment access obstacles. It is imperative that hepatitis C treatment – currently unobtainable for the majority of PWID – is made accessible to all. The rationale for this access must, however, be in relation to the human rights of all to treatment access, rather than in relation to a population-based imperative aimed at disease control.

What difference does this emphasis make? Locating hepatitis C treatment as a human right, rather than a viral eradication issue, acknowledges the fundamental rights and humanity of the most marginalised, including the right of individuals to choose to access treatment – or to decline. It is only on this basis that meaningful and effective community engagement in hepatitis C treatment delivery and advocacy can be mobilised. Such engagement and advocacy have been transformational in regard to HIV treatment access, but have been sorely lacking thus far in the response to hepatitis C.

## A perspective from the European Liver Patients' Association

*By **Margaret Walker** and **Lilyana Chavdarova** of the European Liver Patients Association*.

In the years since its establishment in 2005, the European Liver Patients Association (ELPA) has seen many changes in the way in which liver disease, and particularly viral hepatitis, is perceived. As a result we acknowledge the importance of working in a more holistic manner, ensuring that the wheel is not reinvented over and over again. There is clearly no time to waste! Ignoring the importance and value of experience in other disease areas is both counterproductive and limiting.

The HIV community has a vast amount of experience which has been documented over the years and which is extremely useful to those of us now working in the viral hepatitis arena. A number of lessons can, and should, be taken on board. For example, awareness-raising is one area which warrants a greater focus. Policy-makers, health professionals and the general public are only able to react if they have all of the facts and information in hand. Stigma is an avoidable aspect of viral hepatitis which is strongly linked to lack of knowledge and understanding. The only way to combat it is through education and by giving a face to the disease. In addition to World Hepatitis Day reminding us to “think again,” we need to emphasise to all interested stakeholders that hepatitis C is the 8^th^ biggest killer worldwide yet receives much less attention than other diseases. We should also highlight the fact that people from all walks of life can be, and are, affected by viral hepatitis – the disease does not just touch those who suffer from it but also their immediate and extended families.

Pricing issues are extremely topical at the moment. With new antiviral medications coming into the market, the potential now exists to cure 90% of hepatitis C cases, but pricing is a growing concern. Access to treatment remains one of the key issues for patients, and measures similar to those implemented to tackle the HIV epidemic are needed. These measures could include (but are not limited to) diversified licensing options, large availability of generics and high-volume procurement. In addition, it would be useful to look at the option provided by pricing differentiation, since experience has proven that the current external pricing system needs to be modified to meet the needs of European Union member states advancing at different speeds.

Last but not least, increased efforts to identify hepatitis in high-risk groups, such as those seen for HIV, are greatly needed, as is pinpointing and making use of best or better practices. This is very much linked to affordable and readily available testing for those at risk. Having the different stakeholders concerned working together and using one voice is clearly key to tackling hepatitis C, in particular given the limits placed on all of us by limited resources and capacities.

## Lessons learned from HIV, which can contribute to the global response to hepatitis

*By **Eberhard Schatz**, **Katrin Schiffer**, **John Peter Kools** and **Jason Farell** of Correlation – European Network Social Inclusion and Health*.

The HIV/AIDS movement ensured a high level of awareness, political commitment, programmes and funding through the years, while at the same time the hepatitis C epidemic among people who inject drugs (PWID) has been neglected for more than 20 years. When hepatitis C was addressed in the past, it was solely represented as an HIV/AIDS co-infection, with the specific challenges of hepatitis C ignored.

Tailored hepatitis C prevention, testing, treatment and care interventions are needed. The world is clear about this, as demonstrated by the new hepatitis resolution passed by the World Health Assembly in May 2014. The question now is when such interventions will be implemented broadly in all European countries to the same extent as HIV activities.

Advocates, community members and other stakeholders in the area of hepatitis C should rely on lessons learned from the HIV/AIDS epidemic, and could learn from methods and achievements such as taking a more proactive policy approach both nationally and internationally.

In our opinion, some of the key actions suggested by lessons learned from the three-plus decades of the HIV epidemic are the following:

### General

• Address the economic impact and public health burden. By doing so, the development and implementation of tailored and effective prevention, testing, treatment and care responses become a logical and rational necessity.

• Create a critical mass of urgency and political will. Hepatitis C among PWID is also an issue of stigma and marginalization. These factors cannot only be addressed by doctors and other health professionals.

• Engage different communities to reduce stigma and provide additional support.

• Involve charismatic and popular “influencers” who can create awareness among the general public.

• Through hepatitis C, give a ”face” and therefore a voice to PWID.

• Empower PWID and involve them actively in all advocacy and policy activities.

### Political

• Link universal access to prevention, treatment and health care with an overall human rights agenda.

• Build a case for affordable pricing of effective treatment regimes.

• Advocate for effective prevention, testing, treatment and care programmes at local, regional, national and international levels.

• Ensure a strong commitment from international health organisations including the World Health Organization.

• Advocate for targeted hepatitis C strategies, interventions and action plans, and insist that every country in the world has a national strategy.

### Prevention

• Scale-up comprehensive harm reduction services, including blood-awareness campaigns to ensure required protection.

• Develop and implement interventions to promote safer “route of transmission” campaigns. Alternatives to injecting are effective in the long term.

### Treatment and care

• Establish treatment and care systems consisting of multi-disciplinary teams and integrated services to provide a holistic approach and to ensure that drug users receive all needed support.

• Invest in community-based and other high-impact and low-threshold interventions and care systems.

• Involve family and friends in the development and implementation of family-focused care to support drug users during the hepatitis C treatment phase and to create support for professional services.

## Testimony of an HIV/hepatitis C patient with AIDS and advanced hepatitis C disease

*By **Luís Mendão**, a former drug user and sexually non-orthodox activist and advocate since 1982. Cofounder of G.A.T. – Grupo Português de Activistas sobre Tratamentos de VIH/SIDA – Pedro Santos and member of the European AIDS Treatment Group*.

Hepatitis C affects millions of people. The number of deaths due to liver disease progression will grow if adequate public health policies are not urgently implemented. I am deeply convinced that the involvement of people using drugs and living with hepatitis C in all levels of public policy and programmes to control the epidemic is crucial, so here goes:

I’m Luís Mendão, born in Portugal, 57 years old, heavy nicotine smoker, free from sex and other drugs for a while.

In September 1996, I was taken to the hospital emergency department where a young doctor on duty closed the door and told me that my situation was very serious. I had an infection. My lungs were affected, and I had a severely compromised immune system.

*Luís* – I thought – *you have been gladly promiscuous with many people, male and female, some of them also quite promiscuous, and the truth is that you almost always used condoms, but not always.*

*Luís* – I kept recalling – *you experimented and enjoyed drugs, some injected, a few times. Are you sure you never shared equipment?*

The fear that sex, which was a source of joy and wonder, could have turned into a source of illness and death, was unbearable. If I could share one recommendation it would be related to this anxiety: opt-in for safer sex, and never share injection materials.

We are not obligated to preach abstinence from sex or drugs, but safe sex and not sharing needles are effective in preventing both HIV and hepatitis C transmission. Also, if you had previous risky behaviours, even if only once, get tested for HIV and for hepatitis C. It’s the only way to know if you live with it. You are not obliged to change what you do, but do it more safely, and remember that only by knowing your status can you treat the infections. Treatment exists, and is more and more effective.

I was eventually transferred to the infectious disease ward after confirmation of diagnosis. My laboratory results from September and October 1996 indicated that I had AIDS, a CD4 count of 2, a viral load of 890,000 and terrible liver analysis scores. I figured I would have six to 12 months to live.

I sought information on the disease, to know what to expect. I got a book, well written and clear, that described its progression. My doctor asked me about my expectations. I told him I wanted the rest of the autumn and winter to solve my life, and the spring to say goodbye to the ones I loved. If he could offer me the summer as a bonus, my happiness would be complete. I think he noted it – he notes everything in his beautiful handwriting. He asked me if I felt able to comply with a rigorous and complex treatment. I said yes. I enjoyed myself a lot and organized my days under this health totalitarianism.

The time I had left would be to live, have a loving life and laugh. I decided to tell my friends and lovers (and to get them to test as well), and the majority of my family, but not my father and mother to spare them the suffering. It was difficult waiting for the results of the tests, but one by one my partners’ results came, all negative for HIV.

Two months later I had a CD4 count of 211 and an undetectable viral load.

HIV treatment was already effective in 1996 (though there were difficult side-effects, adverse events and a heavy pill burden). The lucky ones had access. Portugal at the time was on the right side of the planet, triple-combination therapy was accessible, and I was saved.

The summer of 1997 was ending, I had said goodbye to everyone, and death was not coming. I walked the “Santiago Way,” 45 days on foot with a 10 kg backpack, got married, and as money began to fail I went back to work in 1998. I joined the European AIDS Treatment Group in 1999 and founded the Portuguese group GAT – Grupo Português de Ativistas sobre Tratamentos de VIH/SIDA – in 2001.

In 2003, my liver fibrosis was worsening and hepatitis C progression became my major health issue, not AIDS. I tried pegylated interferon and ribavirin – two months of horror and torture, with no results. I was a null responder - intolerant to interferon. I was hospitalized for two months after stopping the treatment. I am a male over the age of 45 with a high body mass index, with diabetes, fatty liver, genotype 1a, a high viral load and later I found out I’m non-CC – the genotype more likely to be cured by HCV treatment. At the time, all were predictors of poor treatment outcomes. But treatment in hepatitis C is changing, and the perspectives are good.

There is much we can learn from our past efforts in the field of HIV. It is imperative, first of all, that responding to the hepatitis C epidemic is part of the public agenda, that both policymakers and communities are aware that the problem exists, and that they understand what can be done to prevent and treat it. A common strategy is essential.

We need to provide treatment to all who require it. This includes active drug users, but also so many more people. Treatment is increasingly effective, but prices are sky-high and issues of affordability rampant.

Integrated care systems to increase adherence and retention in treatment are very important, especially for people with co-morbidities, as well as people in vulnerable social or economic situations. There is a lot more to treatment than just giving people pills.

Promoting access to clinical trials with new treatments for people who lack viable treatment options should also be considered whenever possible. Unlike other kinds of viral hepatitis, there is no vaccine for hepatitis C, making prevention efforts critical, including the provision of safe injection equipment, opioid substitution therapy and safe consumption rooms (for the most affected group). Information on how to avoid transmission and barrier-free HCV counselling, testing and linkage services are also essential to stop the growth of the epidemic.

## Competing interests

The authors declare that they have no competing interests.

## Declarations

This article has been published as part of *BMC Infectious Diseases* Volume 14 Supplement 6, 2014: Viral Hepatitis in Europe. The full contents of the supplement are available online at http://www.biomedcentral.com/bmcinfectdis/supplements/14/S6. The publication charges for this supplement were funded by AbbVie as an unrestricted grant to Rigshospitalet, the University of Copenhagen. AbbVie further funded the printing of the supplement with additional financial support from the Drug Prevention and Information Programme (DPIP) of the European Union. Magdalena Harris was funded by a National Institute for Health Research Post-Doctoral Fellowship (NIHRPDF-2011-04-031).
